# Computational Screening of Metal-Organic Frameworks for Ethylene Purification from Ethane/Ethylene/Acetylene Mixture

**DOI:** 10.3390/nano12050869

**Published:** 2022-03-04

**Authors:** Yageng Zhou, Xiang Zhang, Teng Zhou, Kai Sundmacher

**Affiliations:** 1 Process Systems Engineering, Otto-von-Guericke University Magdeburg, Universitätsplatz 2, D-39106 Magdeburg, Germany; zhouy@mpi-magdeburg.mpg.de (Y.Z.); sundmacher@mpi-magdeburg.mpg.de (K.S.); 2 Process Systems Engineering, Max Planck Institute for Dynamics of Complex Technical Systems, Sandtorstr. 1, D-39106 Magdeburg, Germany; zhangx@mpi-magdeburg.mpg.de

**Keywords:** metal-organic framework, gas separation, ethylene purification, C_2_ hydrocarbons, GCMC simulation, MOF screening

## Abstract

Identification of high-performing sorbent materials is the key step in developing energy-efficient adsorptive separation processes for ethylene production. In this work, a computational screening of metal-organic frameworks (MOFs) for the purification of ethylene from the ternary ethane/ethylene/acetylene mixture under thermodynamic equilibrium conditions is conducted. Modified evaluation metrics are proposed for an efficient description of the performance of MOFs for the ternary mixture separation. Two different separation schemes are proposed and potential MOF adsorbents are identified accordingly. Finally, the relationships between the MOF structural characteristics and its adsorption properties are discussed, which can provide valuable information for optimal MOF design.

## 1. Introduction

Ethylene (C_2_H_4_) is one of the major chemicals in the petroleum industry, which can be produced by thermal cracking from various sources; for instance, natural gas, naphtha, and gasoline [1]. In C_2_H_4_ production, the separations of acetylene (C_2_H_2_) and ethane (C_2_H_6_) from C_2_H_4_ are achieved through catalytic partial hydrogenation and cryogenic distillation, respectively [2]. However, they are very energy-consuming processes due to extreme operating conditions. Over decades, adsorptive separation under mild conditions on a novel class of nano-porous materials, known as metal-organic frameworks (MOF), has been suggested as a substitute [3]. Due to their structural diversities, MOFs feature many outstanding properties such as tunable pore sizes and high surface areas [4], making them ideal for many applications, such as membranes based purification [5], removal of environmental pharmaceutical contaminants [6,7], gas storage, and in particular gas separation.

A number of MOFs have been reported as promising materials for the separation of C_2_H_2_/C_2_H_4_ based on different mechanisms. Some utilize a size-selective sieving effect. For example, Xiang et al. discovered that M’MOF-3a could separate C_2_H_2_ and C_2_H_4_ with a high selectivity of 25.53 at 195 K and 5.23 at 295 K based on the different sizes of the solutes [8]. Hu et al., (2015) revealed that the suitable pores and opening windows of UTSA-100a could lead to a high C_2_H_2_ uptake of 4.27 mmol/g and a C_2_H_2_/C_2_H_4_ selectivity of 10.72 at 296 K and 1.0 bar. Besides, MOFs featuring open metal sites (OMS) have been found effective for the C_2_H_2_/C_2_H_4_ separation due to large affinity. However, the OMS bind strongly with both C_2_H_2_ and C_2_H_4_, resulting in a relatively low selectivity. For instance, Bloch et al. demonstrated that Fe_2_(dobdc) had a high C_2_H_2_ uptake of 6.8 mmol/g, but a low C_2_H_2_/C_2_H_4_ selectivity of 2.08 at 318 K and 1.0 bar [9]. In addition to that, Yang et al. showed that NOTT-300 could separate the mixture through weak supramolecular interactions aligned within the host [10]. Finally, MOFs containing anions (such as SIFSIX) have been also found to be promising for C_2_H_2_/C_2_H_4_ separation [11].

For the separation of C_2_H_4_/C_2_H_6_, there have been many MOFs reported to be C_2_H_4_ selective, such as Fe_2_(dobdc) [9], PAF-1-SO_3_Ag [12], NOTT-300 [10] etc. However, only limited MOFs with reverse selectivities towards C_2_H_6_ have been reported. Gücüyener et al. first demonstrated the C_2_H_6_ selectivity of ZIF-7 and concluded that its selectivity was induced by a gate-opening mechanism [13]. Liao et al. found that MAF-49 had a C_2_H_6_ selectivity of 2.7 and a capacity of 1.73 mmol/g at 298 K [14]. Later, Lin et al. studied Cu(Qc)_2_ and found that its capacity towards C_2_H_6_ was 1.85 mmol/g and the C_2_H_6_/C_2_H_4_ selectivity was 3.4 at 298 K and 100 kPa [15]. Chen et al. reported an iron-based MOF PCN-250 as another C_2_H_6_ selective adsorbent. Its adsorption capacity towards C_2_H_6_ and C_2_H_4_ was 5.21 mmol/g and 4.22 mmol/g at 298 K and 100 kPa, respectively, and the C_2_H_6_/C_2_H_4_ selectivity was in the range of 1.9−4.0 [16].

Unlike the widely investigated binary separations, a direct purification of C_2_H_4_ from the ternary mixture C_2_H_2_/C_2_H_4_/C_2_H_6_ has been much less studied. There have been basically two different strategies. Hao et al. synthesized a MOF TJT-100 that can simultaneously trap C_2_H_2_ and C_2_H_6_, thus leading to high selectivities for C_2_H_2_/C_2_H_4_ of 8.5 and C_2_H_6_/C_2_H_4_ of 5.75 [17]. Chen et al. purified a four-component mixture (C_2_H_2_/C_2_H_4_/C_2_H_6_/CO_2_) in one column packed with three different MOFs with each capturing one impurity [18]. It is worth noticing that both of these novel approaches, where a direct purification of C_2_H_2_ from the ternary C_2_ mixture instead of performing two separate binary separations, can yield intensified process configurations with less unit operations.

To identify candidates with great potential in the direct purification of the ternary C_2_ mixture is a challenging task. Fortunately, the grand canonical Monte Carlo (GCMC) simulation can predict adsorption equilibria fast and efficiently. In fact, it has been tested and proven useful in various gas separations, such as CO_2_/N_2_/CH_4_ [19,20,21], H_2_/CH_4_ [22,23], C_2_H_2_/CH_4_ and C_2_H_2_/CO_2_ [24], C_2_H_6_/C_2_H_4_ and C_2_H_6_/CH_4_ [25], C_2_H_2_/C_2_H_4_ [26], CO_2_/H_2_ [27], C_3_H_8_/C_3_H_6_ [28], hexane and heptane isomers [29], xylene isomers [30], and process-centric CO_2_ capture [31,32].

In this work, we perform a high-throughput screening over 4764 experimentally synthesized MOFs for the separation of C_2_H_2_/C_2_H_4_/C_2_H_6_. First, we validate the accuracy of GCMC simulations by comparing the simulated and experimental uptakes of pure C_2_H_2_, C_2_H_4_, and C_2_H_6_ in a few MOFs. Next, we compute the separation capacity and selectivity for each candidate. Based on that, a new separation performance index (SPI) is defined and discussed in detail. The best MOFs are selected accordingly and the structural characteristics are revealed.

## 2. Computational Details

### 2.1. MOF Database

The computation-ready, experimental metal-organic framework (CoRE MOF) database [33] containing totally 4764 MOF candidates was chosen as the screen basis due to the following reasons. First, this database consists of a variety of MOF structures, which provide a rich search space for finding promising adsorbents; second, the structures in the database are immediately suitable for molecular simulations without any further modifications; third, each MOF has already been experimentally reported and recorded with a unique Cambridge Structural Database (CSD) code [34] so that the screened materials can be synthesized.

### 2.2. Molecular Simulation

Molecular simulation enables multifaceted investigations of intermolecular and intramolecular phenomena on the microscopic scale by advanced computational algorithms. GCMC simulation, with constant chemical potential, volume, and temperature, but variable number of molecules, has been widely used for studying adsorption equilibrium [35]. In this work, we implement GCMC simulations using the software RASPA [36] to estimate the adsorption equilibria of the ternary mixture C_2_H_2_/C_2_H_4_/C_2_H_6_ over the 4764 MOFs.

For the GCMC simulation, various parameters need to be specified properly. First, general parameters such as Monte Carlo moving probabilities, cut-off radius, cell size, and number of cycles are defined. Here, four types of Monte Carlo moves (i.e., translation, rotation, reinsertion, and swap) are considered. The probabilities of the occurrences of these moves are set equal. In addition, a cut-off radius of 12.0 Å is used. The simulation cell size is expanded to at least 24.0 Å along all the three spatial dimensions and the corresponding periodic boundary conditions are applied. Each simulation is carried out with first 30,000 cycles for equilibration and subsequent 20,000 cycles for production. Additionally, the Peng−Robinson equation of state is used to estimate the gas phase fugacities of species.

Next, to calculate the energy state of the whole system, the following force field equation is used [37].

(1) $$U=\sum 4\varepsilon_{ij}\left[{\left(\frac{\sigma_{ij}}{r_{ij}}\right)}^{12}-{\left(\frac{\sigma_{ij}}{r_{ij}}\right)}^{6}\right]+\sum \frac{q_{i}q_{j}}{4\pi \varepsilon_{0}r_{ij}}$$

where *ε_ij_* is well depth, *σ_ij_* is collision diameter, *r_ij_* is the distance between atoms *i* and *j*, *q_i_* is the atomic charge of atom *i*, and *ε*_0_ is 8.8542 × 10^−12^ (C^2^N^−1^m^−2^). The first term describing Van der Waals interactions is the Lennard-Jones (LJ) potential and the second term representing electrostatic interactions is the columbic potential.

In addition to the force field equation, force field parameters are provided. First, for adsorbate molecules (C_2_H_2_, C_2_H_4_, and C_2_H_6_), the Lennard-Jones (LJ) potential parameters (*σ* and *ε*), partial charge (*q*), and bond length (*l*) are specified. Table 1 lists all the molecular parameters of the gas components where those of C_2_H_2_ are taken from [38] and those of C_2_H_4_ and C_2_H_6_ are adopted from [39]. Next, for host frameworks, the LJ potential parameters are adopted from the DREIDING force field [40] and those of the missing atoms are taken from the UFF force field [41]. The charges on the MOF atoms are estimated using the EQeq method [42]. MOFs are considered as rigid and thus the interactions between MOF atoms are ignored. The cross LJ parameters are computed using the Lorentz–Berthelot combining rule [41] except those between Cu of MOF and C of C_2_H_2_ that are modified according to [38].

## 3. Results and Discussion

### 3.1. GCMC Validation

The reliability of GCMC simulations was validated by experimental data. In the literature, the amount of C_2_H_2_ and C_2_H_4_ adsorbed on different MOFs (i.e., MOF-5, ZIF-8, and UTSA-20) has been measured at 298 K and 10^5^ Pa [9,43,44]. In addition, the adsorption uptakes of C_2_H_6_ on Fe-MOF-74, MOF-505, and UTSA-20 have also been measured at 318 K and 10^5^ Pa [43]. Under the same experimental conditions, the pure component uptakes of C_2_H_6_ were simulated and those of C_2_H_2_ and C_2_H_4_ were directly taken from our previous work [26].

**Figure 1:**
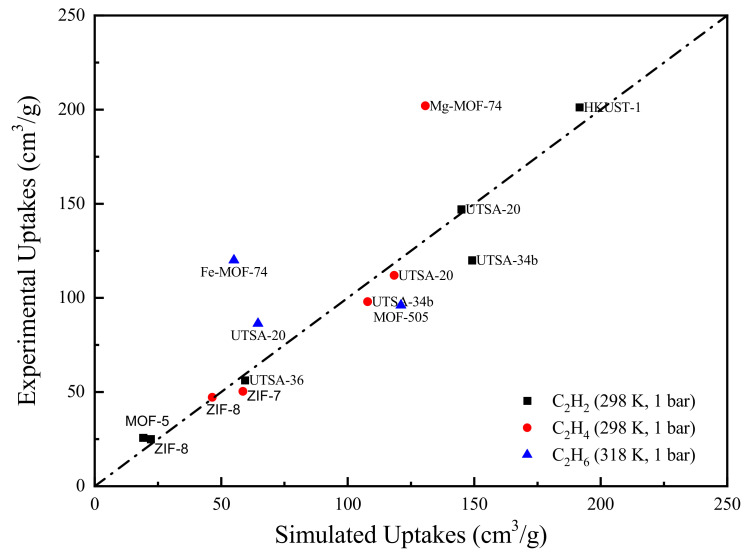
Comparison between experimental and GCMC simulated pure-component adsorption uptakes.

The comparison of the GCMC results and experimental data is presented in Figure 1. Clearly, the majority of the data lies close to the parity line, indicating an overall good agreement between experimental and simulated uptakes. However, some MOFs such as Mg-MOF-74 and Fe-MOF-74 lie far away from the parity line. This is mainly due to the fact that the M-MOF-74 (where M=Mg, Fe et al.) family contains open metal sites (OMS), which can strongly bind with the adsorbates [45]. In this case, the standard force field parameters are unable to appropriately capture these interactions. For better illustration, we calculated the zero coverage isosteric heat of adsorption $Q_{st}^{0}$ for the two outliers. Note that the absolute value of $Q_{st}^{0}$ indicates the strength of the MOF-adsorbate interactions. As shown in Table 2, the two $Q_{st}^{0}$ derived from molecular simulation are significantly lower than the corresponding experimental values, which demonstrates the lack of accurate force field parameters for the description of coordination interactions between OMS and adsorbates. Some methods such as quantum mechanics (QM) calculations [46,47] have been developed to improve the accuracy of force field parameters. However, the identification of MOFs containing OMS is very laborious and the re-adjustment of force field parameters through rigorous QM calculations is computationally expensive. Therefore, to facilitate the large-scale MOF screening, we adopted the general force field parameters to keep a compromise between simulation precisions and computational cost.

### 3.2. Capacity and Selectivity

For the evaluation of separation performance of adsorbent, the capacity and selectivity are two important indicators. The adsorption capacity $q_{i}$ (i = C_2_H_2_, C_2_H_4_ and C_2_H_6_) is usually defined as the amount of gas adsorbed in the solid adsorbent. Additionally, the selectivity is typically defined for a binary system (component = *i*, *j*):

(2) $$S_{i/j}=\frac{K_{i}}{K_{j}}=\frac{x_{i}}{y_{i}}/\frac{x_{j}}{y_{j}}$$

where *K_i_* is the equilibrium constant. *y_i_* and *x_i_* are the molar fractions of species *i* in the gas and solid phases, respectively. In a ternary system, selectivity can be defined likewise. For instance, the task of this work is to separate C_2_H_2_ and C_2_H_6_ from the ternary mixture in order to obtain a purified C_2_H_4_ product. Obviously, MOFs with high C_2_H_2_ and C_2_H_6_ uptakes and low C_2_H_4_ uptake are desired. Thus, two selectivity indicators ($S_{C_{2}H_{2}/C_{2}H_{4}}$ and $S_{C_{2}H_{6}/C_{2}H_{4}}$) can be defined for MOF screening where the first is the selectivity of C_2_H_2_ over C_2_H_4_ and the second is the selectivity of C_2_H_6_ over C_2_H_4_ in the ternary mixture.

**Figure 2:**
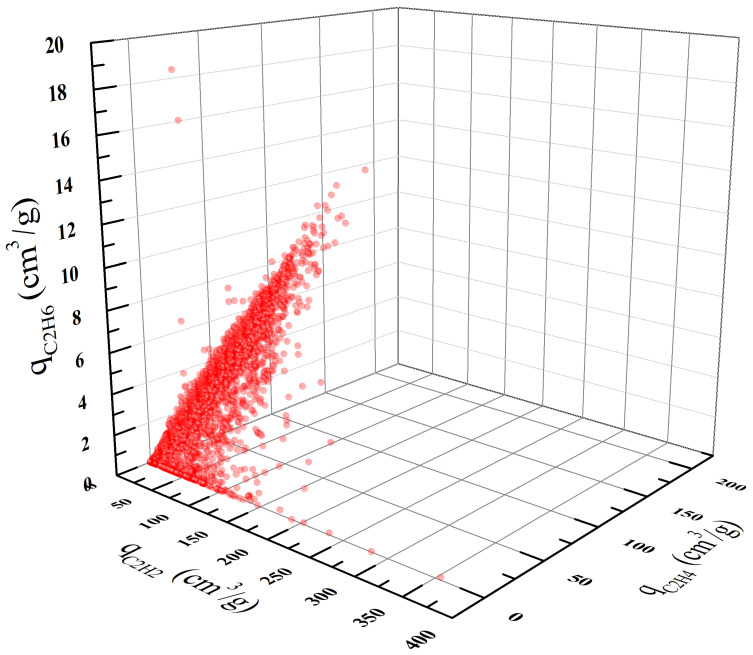
Equilibrium adsorption uptakes of C_2_H_2_, C_2_H_4_, and C_2_H_6_ in the ternary mixture at 298 K and 1 bar.

We conducted GCMC simulations for all the 4764 MOFs in the CoRE MOF database. The concentrations of the three components C_2_H_2_/C_2_H_4_/C_2_H_6_ were set to 5.0/90/5.0 (mol/mol/mol), and the adsorption simulation was conducted at ambient conditions (i.e., 298 K and 1.0 bar). Note that only 4462 of the 4764 MOFs show valid non-zero uptakes. The obtained equilibrium adsorption loadings of the 4462 MOFs are plotted in Figure 2 with each point representing a single MOF. From this figure, we found that the adsorption uptakes of the three components are roughly in the order of q_C2H2_ > q_C2H4_ > q_C2H6_. This can be explained by the differences in the molecular model parameters. As indicated in Table 1, for both C_2_H_6_ and C_2_H_4_ we used two-site models with three parameters. In contrast, C_2_H_2_ was modeled as a four-site molecule with additional point charge parameters. The introduction of point charges on the C and H atoms provides additional electrostatic interactions between C_2_H_2_ and the MOF atoms, which leads to the highest adsorption uptake of C_2_H_2_. Furthermore, a strong correlation between the uptakes of C_2_H_4_ and C_2_H_6_ can be observed from Figure 2. This is primarily due to their similar molecular models and parameters (see Table 1).

**Figure 3:**
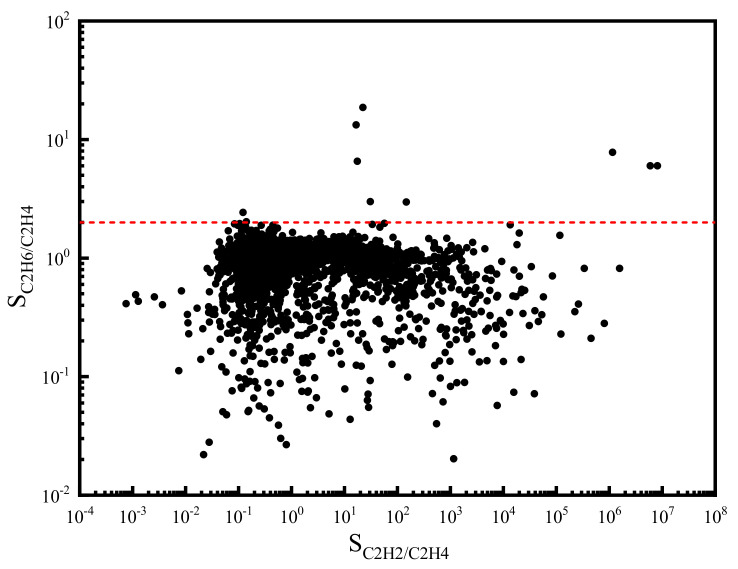
Adsorption selectivity of C_2_H_2_/C_2_H_4_ and C_2_H_6_/C_2_H_4_ in the ternary mixture at 298 K and 1 bar (The red dash line: S_C2H6/C2H4_ = 2.0).

In addition to the adsorption capacity, we calculated the separation selectivity of C_2_H_6_/C_2_H_4_ and C_2_H_2_/C_2_H_4_ for each MOF candidate. The results are shown in Figure 3. It can be found that the selectivity of C_2_H_2_/C_2_H_4_ spans a wide range. By contrast, the distribution of C_2_H_6_/C_2_H_4_ selectivity is much narrower. Moreover, most of the C_2_H_6_/C_2_H_4_ selectivity is lower than 2.0 (red dash line), which reveals the relative difficulty for the separation of C_2_H_6_ from C_2_H_4_.

### 3.3. Selection of Potential MOFs

The product of selectivity and capacity has been widely used as a criterion for the selection of MOFs for binary gas separations [24,48]. However, this simple performance indicator needs some modifications before it can be used to rank MOF candidates for ternary separation systems. First, unlike binary systems where a unique pair of selectivity and capacity is involved, for ternary mixtures, selectivity and capacity must be carefully defined to give an appropriate description of the separation performance. Second, the selectivity and capacity often differ in several orders of magnitude. Thus, the direct product of selectivity and capacity can be dominated by one single factor. Such a biased metric is not useful for the screening of best adsorbents. Based on these considerations, we propose a new selection performance indicator (SPI) for the evaluation of the performance of MOFs for the separation of the ternary C_2_H_2_/C_2_H_4_/C_2_H_6_ mixture.

(3) $${SPI=log(S}_{C_{2}H_{2}{/C}_{2}H_{4}}{)\;\times \;q}_{C_{2}H_{2}}{\;\times \;log(S}_{C_{2}H_{6}{/C}_{2}H_{4}}{)\;\times \;q}_{C_{2}H_{6}}$$

(4) $${SPI}_{C_{2}H_{2}}{=log(S}_{C_{2}H_{2}{/C}_{2}H_{4}}{)\;\times \;q}_{C_{2}H_{2}}$$

(5) $${SPI}_{C_{2}H_{6}}{=log(S}_{C_{2}H_{6}{/C}_{2}H_{4}}{)\;\times \;q}_{C_{2}H_{6}}$$

**Figure 4:**
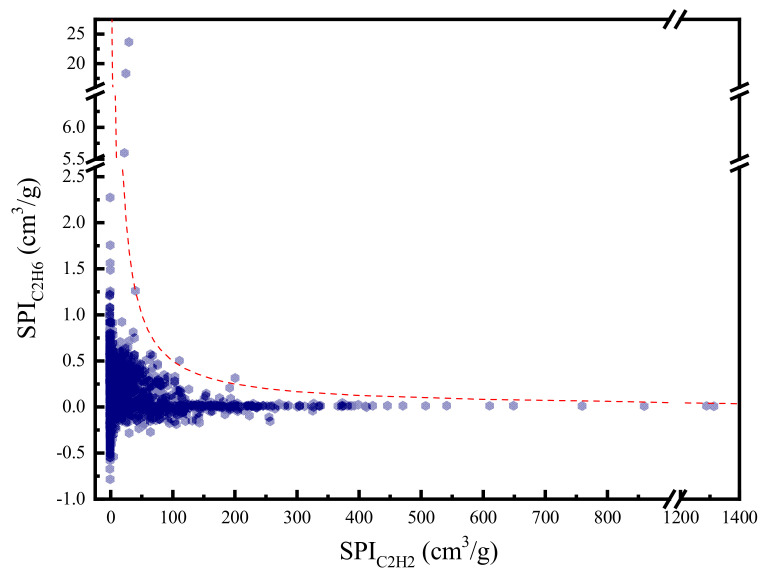
C_2_H_2_/C_2_H_4_ separation performance versus C_2_H_6_/C_2_H_4_ separation performance (The red dash line: SPI = 50).

To provide a deeper insight of the selection metrics, we divide the SPI into two parts. The product of the first two terms denoted as ${SPI}_{C_{2}H_{2}}$ represents the performance of MOF for C_2_H_2_ separation. Similarly, the product of the last two terms denoted as ${SPI}_{C_{2}H_{6}}$ measures MOF’s performance for C_2_H_6_ separation. Figure 4 plots the ${SPI}_{C_{2}H_{2}}$ and ${SPI}_{C_{2}H_{6}}$ for all the MOF candidates. It can be observed that ${SPI}_{C_{2}H_{6}}$ is generally much smaller than ${SPI}_{C_{2}H_{2}}$. This confirms that the separation of C_2_H_6_ from C_2_H_4_ is more difficult than the separation of C_2_H_2_ from C_2_H_4_. Moreover, the distribution of ${SPI}_{C_{2}H_{2}}$ and ${SPI}_{C_{2}H_{6}}$ forms a Pareto-like front, which indicates a competitive relation between the separation of C_2_H_2_ and C_2_H_6_.

**Figure 5:**
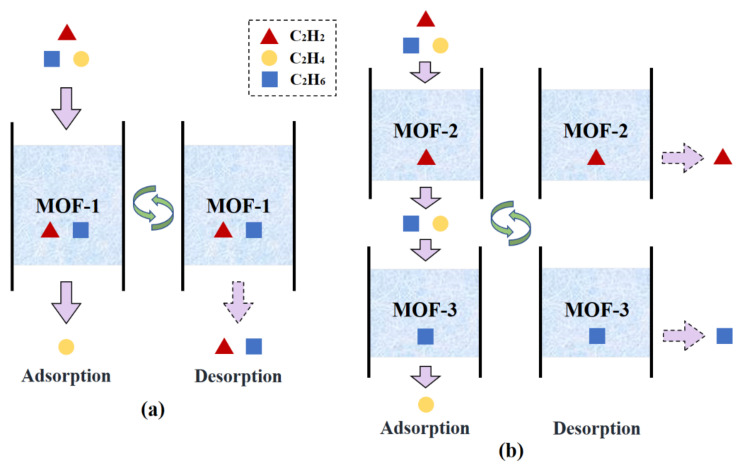
Schematic diagram of two adsorption process configurations for the separation of the C_2_H_2_/C_2_H_4_/C_2_H_6_ mixture: (**a**) single-step separation; (**b**) multi-step separation.

Two different separation schemes are proposed for the ternary mixture: i.e., single-step separation and multi-step separation. As illustrated in Figure 5a, in the single-step separation process, C_2_H_2_ and C_2_H_6_ are adsorbed simultaneously in a single sorption cycle and a pure C_2_H_4_ product stream can be obtained directly. This separation process uses only one adsorbent material, which significantly reduces the process complexity. Accordingly, the MOF selection criterion is the maximization of SPI that compromises the material performance for both C_2_H_2_ and C_2_H_6_ separations. Table 3 lists the best 10 MOFs for the single-step separation as well as their corresponding structural properties, selectivity, capacity, and SPI values. As depicted, the most promising MOF is CUNXIS, showing a maximal SPI of 705.8 cm^6^/g^2^.

Although the single-step process is easy to operate, it may be difficult to find an adsorbent that shows excellent separation performance for both C_2_H_2_ and C_2_H_6_. Additionally, the regeneration of MOF produces a C_2_H_6_/C_2_H_2_ mixture, which needs to be further separated and recycled back to the cracking reactor for maximizing the C_2_H_4_ yield. Considering these factors, another multi-step separation process shown in Figure 5b is introduced. Unlike the single-step separation, the multi-step process separates each impurity on an individual adsorbent sequentially. For purifying C_2_H_4_ from C_2_H_2_/C_2_H_4_/C_2_H_6_, two different strategies can be employed. Specifically, one can first select a C_2_H_2_-selective MOF to separate C_2_H_2_ and then employ another C_2_H_6_-selective MOF to adsorb C_2_H_6_. Alternatively, the opposite separation sequence can also be applied. The top five C_2_H_2_-selective MOFs and C_2_H_6_-selective MOFs are listed in Table 4 according to their individual SPI values. The numerous combinations of one adsorbent from each group provide a big chance for the successful implementation of the multi-step separation process. For example, out of all the 25 combinations, ORAQUU and CUNXIS can be selected due to their highest ${SPI}_{C_{2}H_{2}}$ and ${SPI}_{C_{2}H_{6}}$ values.

### 3.4. Structure-Property Relationship Study

**Figure 6:**
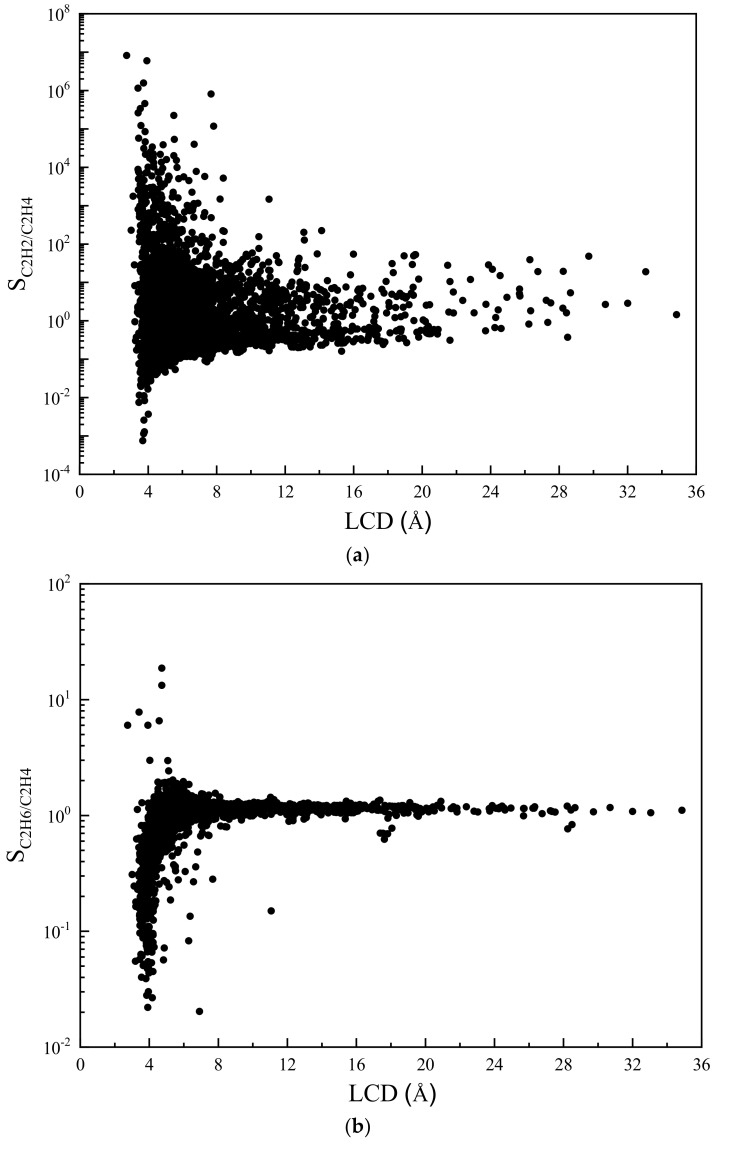
Adsorption selectivity of MOF with respect to (**a**) C_2_H_2_/C_2_H_4_ and (**b**) C_2_H_6_/C_2_H_4_ versus LCD.

Some valuable insights can be extracted from the screening results by conducting the structure–property relationship study. These insights provide useful information for the experimental design of novel high-performing adsorbents. First, to quantify the influence of structural parameters on the adsorption selectivity, the relationship between the largest cavity diameter (LCD) and the selectivity of C_2_H_2_/C_2_H_4_ and C_2_H_6_/C_2_H_4_ is shown in Figure 6. As depicted in Figure 6a, the majority of the C_2_H_2_/C_2_H_4_ selectivity are higher than 1.0 and the highest selectivities are generally achieved at very low LCDs (below 4.0 Å). When only looking at the data, the C_2_H_2_/C_2_H_4_ selectivity of which is above 1.0, the selectivity generally decreases as the LCD increases. Similar trends in relations between LCD and selectivity were reported in the literature [21,49]. In Figure 6b, however, one can see that the selectivity of C_2_H_6_/C_2_H_4_ increases with LCD and reaches its highest value at around 4.7 Å. As the LCD increases further, both selectivities tend to converge to 1.0. This indicates that MOFs with very large LCDs are neither C_2_H_2_-selective nor C_2_H_6_-selective. This is not surprising because when the LCD is small, where three components have to compete for limited adsorption space, molecules with smaller size will be bound more easily. When LCD increases, more adsorption sites become available, hence all the components eventually will have equal chances to be adsorbed on frameworks.

**Figure 7:**
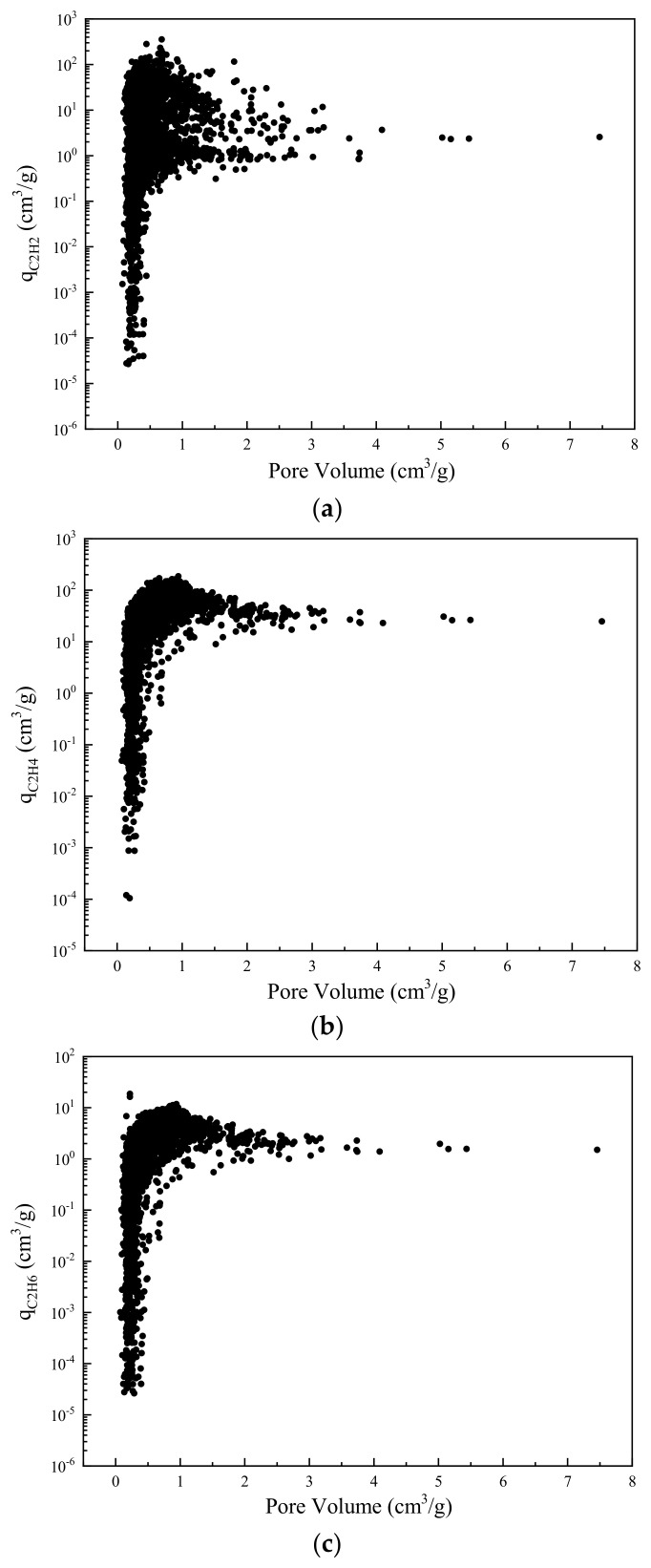
Adsorption capacity of MOF for (**a**) C_2_H_2_, (**b**) C_2_H_4_, and (**c**) C_2_H_6_ with dependence on pore volume.

Figure 7 shows the dependency of adsorption capacity on pore volume of MOF. As indicated in Figure 7a, the adsorption capacity of C_2_H_2_ increases with the pore volume until it achieves a maximal value of 354.7 cm^3^/g at pore volume of 0.68 cm^3^/g. As the pore volume increases further, the capacity of C_2_H_2_ starts to decline and finally converges to 2.5 cm^3^/g. Figure 7b,c shows a similar trend. Generally, the adsorption capacities of both C_2_H_4_ and C_2_H_6_ increase with pore volume until reaching the peak. Further increasing the pore volume, the adsorption capacities of C_2_H_4_ and C_2_H_6_ gradually decline to a limit value of 25.0 cm^3^/g and 1.5 cm^3^/g, respectively. It is observed that high capacities are achieved at moderate pore volumes. This might be explained by the fact that the pore volume is inversely proportional to framework density as demonstrated by Kong et al. [50]. Thus, adsorption capacities are always subjected to a balance between pore volume and MOF density. Comparing Figure 7a–c, although increasing the pore volume until 0.68 cm^3^/g leads to a larger adsorption capacity of impurities C_2_H_2_ and C_2_H_6_, it also causes a higher loss of product C_2_H_4_. Besides, due to the very analogous dependency of adsorption capacities of C_2_H_4_ and C_2_H_6_ on pore volume, the amount of removed C_2_H_6_ is always proportional to the amount of lost C_2_H_4_.

## 4. Conclusions

In summary, we studied the separation of C_2_H_4_ from the C_2_H_2_/C_2_H_4_/C_2_H_6_ mixture and screened the MOF adsorbents from the CoRE MOF database by GCMC simulation. We first validated its accuracy and showed that the force field in general yielded satisfactory results for adsorption equilibrium prediction, except for a few MOFs with the OMS effect. Next, new evaluation metrics SPI were proposed for single-step and multi-step separation strategies for C_2_ ternary mixtures. For single-step separation processes, among all, CUNXIS with the highest SPI value at 705.8 cm^6^/g^2^ was identified as the best. For multi-step separation processes, ORAQUU and CUNXIS with SPI being 1318.5 cm^3^/g and 23.6 cm^3^/g, respectively formed the best combination. Finally, we discovered that S_C2H2/C2H4_ decreased with increasing LCD, and S_C2H6/C2H4_ increased with increasing LCD. For all three components, capacities increased first with pore volume until reaching the peak and after that slowly converged.

However, there are two drawbacks of our study. First, we assumed that thermodynamics is the dominant controlling mechanism in our adsorption system, hence kinetics are ignored. Consequently, MOFs that achieved separations by exploiting the differences in diffusion rates of different species were out of the scope of the search. Second, the selection criteria SPI may not necessarily guarantee a success in practical processes, because it is derived from the phase level properties alone. So future studies containing detailed process modelling or experiments can be carried out for improvement.

## Figures and Tables

**Table 1:** Molecular Parameters of C_2_H_2_, C_2_H_4_, and C_2_H_6_.

	Atoms	*σ* (Å)	*ε* (K)	*q* (e)	Bond Length *l* (Å)
C_2_H_2_	C in C_2_H_2_	3.800	57.875	−0.278	1.211 (C≡C)
	H in C_2_H_2_	0	0	0.278	1.071 (C—H)
C_2_H_4_	CH_2_ (sp^2^)	3.685	93.0	0	1.330 (C=C)
C_2_H_6_	CH_3_ (sp^3^)	3.750	98.0	0	1.540 (C—C)

**Table 2:** Isosteric heat of adsorption at zero coverage $Q_{st}^{0}$.

	$\mathrm{Simulated}\;Q_{st}^{0}\;(\mathrm{kJ}/\mathrm{mol})$	$\mathrm{Experimental}\;Q_{st}^{0}\;(\mathrm{kJ}/\mathrm{mol})$
C_2_H_6_ in Fe-MOF-74 at 318 K	−20.1	−28.2
C_2_H_4_ in Mg-MOF-74 at 298 K	−22.8	−43.0

**Table 3:** Structural properties, selectivity, capacity, and SPI of the top 10 MOF candidates for the single-step separation of C_2_H_2_/C_2_H_4_/C_2_H_6_.

Rank	CSD Code	Metal	LCD (Å)	Pore Volume (cm^3^/g)	$S_{C_{2}H_{2}{/C}_{2}H_{4}}$	$q_{C_{2}H_{2}}$ (cm^3^/g)	$S_{C_{2}H_{6}{/C}_{2}H_{4}}$	$q_{C_{2}H_{6}}$ (cm^3^/g)	SPI (cm^6^/g^2^)
1	CUNXIS	Al	4.73	0.22	22.2	22.2	18.7	18.6	705.8
2	CUNXIS10	Al	4.73	0.22	16.6	20.4	13.3	16.3	455.0
3	GIHBII	Ga	4.58	0.17	17.5	18.3	6.56	6.85	127.4
4	NEXXEV	Li	10.14	0.92	36.7	128.8	1.18	4.15	60.8
5	JAVTAC	Al	5.08	0.21	147.4	51.5	2.97	1.04	54.7
6	BEKSAM	Ga	4.04	0.13	30.8	27.1	2.99	2.63	50.4
7	XEDPON	Zn	7.48	0.56	48.0	114.6	1.17	2.80	37.6
8	LEVNOQ01	Mg	5.91	0.58	16.4	56.1	1.32	4.52	37.0
9	XEKCAT01	Mg	5.92	0.67	11.5	61.1	1.22	6.50	36.4
10	EYACOX	Eu	8.14	0.72	15.3	64.5	1.23	5.17	34.9

**Table 4:** Structural properties, selectivity, capacity, and SPI of the top five MOFs for C_2_H_2_ separation (ranked according to $SPI_{C_{2}H_{2}}$ ) as well as the top five MOFs for C_2_H_6_ separation (ranked according to $SPI_{C_{2}H_{6}}$ ) for the multi-step separation of C_2_H_2_/C_2_H_4_/C_2_H_6_.

Rank	CSD Code	Metal	LCD (Å)	Pore Volume (cm^3^/g)	$S_{C_{2}H_{2}{/C}_{2}H_{4}}$	$q_{C_{2}H_{2}}\;({\mathrm{cm}}^{3}/g)$	$SPI_{C_{2}H_{2}}\;({\mathrm{cm}}^{3}/g)$
1	ORAQUU	Bi, Zn	8.39	0.68	5216.4	354.7	1318.5
2	FENVOL	Zn	6.69	0.44	39528.8	281.4	1293.7
3	ZUQVIQ	Mn	5.78	0.66	5033.4	232.8	861.6
4	OHOFEW	Co	7.31	0.68	5735.7	202.6	761.5
5	VEHNED	Na, Ni	3.81	0.22	454736.8	115.0	650.5
**Rank**	**CSD Code**	**Metal**	**LCD (Å)**	**Pore Volume (cm^3^/g)**	$S_{C_{2}H_{6}{/C}_{2}H_{4}}$	$q_{C_{2}H_{6}}\;({\mathrm{cm}}^{3}/g)$	$SPI_{C_{2}H_{6}}\;({\mathrm{cm}}^{3}/g)$
1	CUNXIS	Al	4.73	0.22	18.7	18.6	23.6
2	CUNXIS10	Al	4.73	0.22	13.3	16.3	18.3
3	GIHBII	Ga	4.58	0.17	6.56	6.85	5.6
4	UFATEA01	Ni	5.37	0.42	2.02	7.41	2.3
5	CEYPUT	Co	5.37	0.42	1.82	6.71	1.7

## Data Availability

Not applicable.
